# “They’re not mentally ill, their lives are just shit”: Stakeholders’ understanding of deaths of despair in a deindustrialised community in North East England

**DOI:** 10.1016/j.healthplace.2024.103346

**Published:** 2024-09-08

**Authors:** Timothy Price, Victoria McGowan, Shelina Vishram, John Wildman, Clare Bambra

**Affiliations:** a Population Health Sciences Institute, Faculty of Medical Sciences, Newcastle University, Newcastle Upon Tyne, NE1 4LP, UK; b Newcastle University Business School, Frederick Douglass Centre, Helix Science Square, Newcastle University, Newcastle Upon Tyne, NE4 5TG, UK

**Keywords:** Deaths of despair, Mental health, United Kingdom, Deindustrialisation, Structural violence

## Abstract

The rise in mortality in high-income countries from drug, suicide, and alcohol specific causes, referred to collectively as ‘deaths of despair’, has received growing interest from researchers. In both the US and UK, mortality rates from deaths of despair are higher in deprived, deindustrialised communities. In this qualitative study, we sought to learn how stakeholders working with vulnerable populations in Middlesbrough, a deindustrialised town in North East England with above average mortality from deaths of despair, understand and explain the prevalence of deaths from these causes in their area. Participants identified a number of structural and socio-cultural determinants that they believe drive deaths of despair in their community, including the effects of austerity, deindustrialisation, communal identity, and collective trauma; we argue that these determinants are themselves a product of structural violence.

## 1 Introduction

Since the early 1900s, life expectancy has generally risen in high-income countries (Ho and Hendi, 2018). While many high-income countries saw a decline in life expectancy in 2014–15, most resumed the trend of increasing life expectancy in 2015–16, with the US and UK being two notable exceptions (Ho and Hendi, 2018). Economists Anne Case and Angus Deaton observed that the decline in US life expectancy was attributable to increasing mortality rates for 45–54-year-old non-Hispanic whites between 1999 and 2013; an increase that was driven primarily by drug overdoses, suicide, and alcohol-specific (DSA) mortality (Case and Deaton, 2015, 2017, 2020). Case and Deaton proposed that worsening labour market conditions (among other factors including rising age at marriage, increased childcare responsibilities, and low educational attainment) caused cumulative disadvantage from one birth cohort to the next that has given rise to a sense of despair and ultimately increased the likelihood of DSA mortality (Case and Deaton, 2017).

Since Case and Deaton coined the term deaths of despair, researchers around the world have adopted this phrase and investigated DSA mortality in different settings. While the increase in DSA mortality in the US was initially regarded as atypical for high-income countries, recognition of increasing mortality from these causes has driven research interest in the UK (Leon et al., 2019; Walsh et al., 2021; Augarde et al., 2022; Camacho et al., 2024; Dowd et al., 2023). While the available evidence suggests that the stagnation in life expectancy in the UK before the COVID-19 pandemic was only partially attributable to DSA mortality (Ho and Hendi, 2018), drug overdoses in men have increased substantially in England since 2012 (Hiam et al., 2017; ONS, 2023a), and suicide mortality has been rising in the UK since 2016 (Augarde et al., 2022; ONS, 2023c; Dowd et al., 2023). The evidence base surrounding DSA mortality in the UK is largely consistent with that in the US, where these deaths have risen significantly since the early 2000s and have primarily affected people of middle-age (Case and Deaton, 2015, 2017). Increases in DSA mortality in both the UK and the US have been primarily driven by significant increases in drug-related mortality, with alcohol-specific mortality and deaths by suicide increasing at a more moderate rate (Augarde et al., 2022; Dowd et al., 2023). Within England specifically, northern regions experience a significantly higher burden of DSA mortality relative to other regions (Augarde et al., 2022; Camacho et al., 2024).

According to the latest available data, the North East has the highest rate of DSA mortality of all English regions (ONS, 2023a; ONS, 2023c). In 2022, Middlesbrough was the local authority with the highest rates of drug-related mortality and alcohol-specific mortality and the second highest rate of suicide in the North East (ONS, 2023a; ONS, 2023c; ONS, 2024a). Middlesbrough is a former industrial town of 143,900 people in the Teesside area of North Yorkshire, England (ONS, 2023b). Once an economic powerhouse that was central to the accumulation of British capital in the 20th century, Middlesbrough experienced rapid deindustrialisation in the latter half of the century that ushered in widespread unemployment and resulting deprivation (Hudson, 1986, 2005; Telford and Lloyd, 2020). A detailed overview of Middlesbrough’s economic history is available as supplemental material. Teesside communities like Middlesbrough are home to some of the most pronounced health inequalities in the country (Bambra, 2019; Mattheys et al., 2016). In Middlesbrough, life expectancy is shorter, rates of smoking are higher, and more people are living with a disability that severely impacts their day-to-day life than in other areas of England (ONS, 2023b; PHE, 2020). Middlesbrough has the highest proportion of severely deprived neighbourhoods of any community in England and the highest proportion of children living in poverty (IOD2019, 2019). These wealth and health inequalities are deeply entrenched and have widened in recent years, leading to the rise of what have been termed ‘left behind’ communities (Bambra et al., 2018; Bolton et al., 2019).

While the inequalities present in Middlesbrough are striking, they are not unique among deindustrialised places. Deindustrialised communities in the US, such as Flint, Michigan, are also home to stark health inequalities and widespread deprivation (Allgood et al., 2022; Henderson et al., 2023). Former industrial regions, such as the US Rust Belt, experience significantly higher rates of DSA mortality than other US regions (Chetty et al., 2016; Herzog, 2020), and similar patterns in DSA mortality have been observed in former industrial areas in Eastern Europe (King et al., 2022; Stuckler et al., 2009). The high level of deprivation, above-average rates of DSA mortality, and similarity to other deindustrialised communities experiencing disproportionate rates of DSA mortality make Middlesbrough a suitable case-study site for investigation into how stakeholders working with vulnerable populations understand and explain the determinants of DSA morbidity and mortality in their community. Despite growing academic interest in DSA mortality, to our knowledge, having reviewed the available literature, no qualitative research investigating stakeholder perceptions of DSA mortality in the UK has yet been published.

## 2 Methods and theoretical framework

### 2.1 Theoretical framework

The concept of structural violence is a useful theoretical framework and a novel approach through which to approach the determinants of DSA mortality. Structural violence has its roots in the field of peace studies; an interdisciplinary field that draws on elements of history, sociology, political science, philosophy, and others. Structural violence is a concept that explains that political and economic systems and policies in a society can perpetuate violence and cause significant harm to individuals or groups of vulnerable people (Galtung, 1969). While the term ‘violence’ recalls images of physical conflict or warfare, Galtung (1969) defined it as the difference between the potential human condition, where one’s fundamental human needs could be met, and the actual human condition, where one’s basic needs are not met. Building on this definition, Banerjee et al. (2012) describes structural violence as “the role that institutions and social practices play in preventing people from meeting their basic needs or realizing their potential” (p.390). Importantly, how structural violence operates is context-specific and varies significantly from place to place. The spatial aspect of structural violence underscores that the specific political, economic, and social contexts of a region or community shape the forms and impacts of violence experienced by people living there. Structural violence has been previously used to examine the communal harm and ill-health that result from the process of, and government responses to deindustrialisation (Clark, 2023; High, 2021; Telford and Lloyd, 2020).

Structural violence is somewhat related to the concept of the social determinants of health, which describes how societal, economic, and political structures impact individuals’ health outcomes (Bartley, 2016). Both structural violence and the social determinants of health seek to identify the upstream forces that set the stage for ill-health. While there are clear commonalities between these two concepts, the framing of health inequalities as *violent* is evocative and facilitates a direct reconciliation with the fact that the structures that govern modern life cause very real harm. Structural violence’s focus on the *structural* causes of ill-health, rather than behavioural causes, is of particular significance in the context of this research, particularly surrounding the issues of drug and alcohol addiction. Research examining the neurobiological causes and indicators of addiction has, thus far, failed to meaningfully advance treatments for addiction (Satel and Lilienfeld, 2013; Sinha et al., 2011). One reason for the failure of neurobiology to develop effective strategies to treat addiction may be that research has neglected the role of social forces, such as social exclusion and the marginalization of people living with addiction (Heilig et al., 2016). Similarly, biomedical models of mental health have been criticised for their failure to account for the contribution of social conditions to creating mental ill-health (Bentall, 2010; Deacon, 2013). Marginalized communities facing spatial and structural inequalities are often unjustly targeted and stigmatized in popular and policy discourse, reinforcing segregation and leading to the pathologization of their residents (Wacquant, 2007; Ward et al., 2017). Wacquant (2007, p.67) describes this as the “blemish of place”, which stigmatises residents based on race, class, and location. Using structural violence as a theoretical framework through which to approach the issues of DSA mortality in a specific place could generate critical insights into what social structures and policy decisions contribute to this marginalization and drive mortality from these causes. In conducting this study, we sought to learn how stakeholders working with vulnerable populations in Middlesbrough understand and explain the determinants of deaths from these causes in their area.

### 2.2 Recruitment

**Fig. 1:**
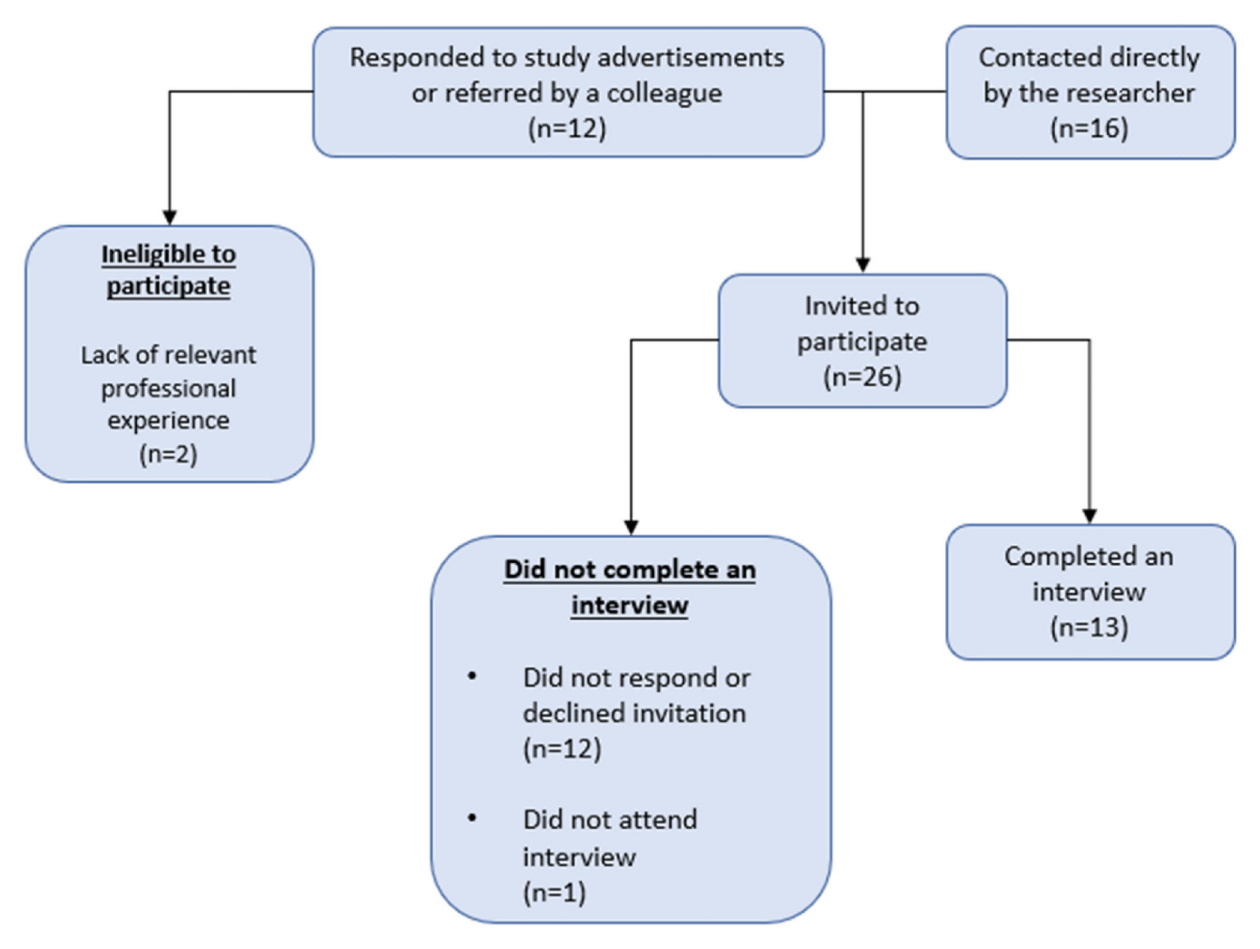
Recruitment flowchart.

Stakeholders were eligible to participate in this study if their work involved people living in Middlesbrough and pertained directly or indirectly to DSA morbidities and mortalities. Recruitment began in September 2022 and concluded in December 2022, at which point we deemed that data saturation had been reached. Several purposive sampling techniques were used in recruitment for this study. A short description of the study and an invitation to participate was distributed through a mailing list for stakeholders in Teesside. Individual stakeholders were also approached directly via email and invited to participate. Stakeholders were approached directly if they worked in a sector that had been frequently discussed in previous interviews but was as yet unrepresented in the sample; for example, multiple participants discussed the role that town councillors have in setting policies that affect people living in deprived areas, so town councillors from particularly deprived wards were emailed directly and invited to participate. Snowball sampling of participants’ networks was used to further the reach of recruitment materials. Snowball sampling is an appropriate recruitment technique when the target population is difficult to access, as was the case given the researchers’ limited existing connections to Middlesbrough stakeholders (Naderifar et al., 2017). Fig. 1 outlines the recruitment process.

### 2.3 Data collection

Thirteen participants completed an interview. Data were collected through semi-structured, in-depth interviews. Interviews were guided by a bespoke topic guide (see supplemental material). The topic guide was informed by a review of the existing literature around DSA morbidity and mortality and was loosely modelled after a discussion guide previously used in a qualitative study investigating communal perceptions of diseases of despair in the US (George et al., 2021). The topic guide used during all interviews has been included as supplemental material. While our review of the literature prior to undertaking data collection suggested that structural violence would be an appropriate theoretical framework through which to analyse our data, this was not communicated to participants so as to avoid influencing them to think exclusively about structural determinants during their interviews. Instead, participants were asked about how a range of factors, from individual mental health to socioeconomic deprivation, may contribute to deaths of despair. We also did not use the term ‘deaths of despair’ when speaking to participants or advertising the study, instead preferring the more neutral “Drug, Suicide, and Alcohol-Specific” morbidity and mortality.

Interviews were conducted online using Microsoft Teams (n = 2), inperson at the participants’ places of work (n = 9), or in a public setting such as a café or coffee shop (n = 2) according to participants’ preferences. Stakeholders worked in a diverse range of professional backgrounds including law enforcement, charity service provision (such as foodbanks and homeless outreach), mental health treatment, substance abuse recovery support, community organising, local government, public health, and housing management. Gathering diverse stakeholder views elicits valuable nuance and multiple insights by incorporating a broad range of perspectives and expertise, which enhances the depth and comprehensiveness of the findings. Table 1 provides demographic information for participants that was collected via a short survey prior to starting the interview.

### 2.4 Ethical considerations

This study was deemed ethically low-risk and approved by the Newcastle University Faculty of Medical Science Research Ethics Committee (Ref: 22812/2022). Participants were provided with a participant information sheet describing the purpose of the study and given the opportunity to ask questions before agreeing to an interview. Prior to the start of their interview, participants provided written consent to take part in the study. Interviews were audio recorded, transcribed, and anonymised by the lead author (TP). Audio recordings of the interviews were deleted once a complete anonymised transcript had been generated. Illustrative quotes included in the Findings and Discussion section of this paper are not attributed to specific participants to mitigate the risk of reconstruction of specific participant transcripts.

### 2.5 Data analysis

Data analysis was conducted using the Iterative Categorization (IC) technique developed by Neale (2016). IC is a technique for analysing qualitative data that has previously been used to support research investigating addiction (Neale et al., 2012, 2017). Coding was conducted using the qualitative analysis software MAXQDA 2022 (VERBI Software, 2022). An initial coding matrix was generated deductively based on the interview topic guide; codes were merged, and the matrix was supplemented with codes generated inductively as coding progressed. Participant narratives identified a number of structural factors as increasing the risk of deaths of despair and these were used to generate codes related to structural violence; these codes were in turn used to identify the specific policies and time periods participants identified as driving DSA morbidity and mortality. Once coding was completed, analysis followed the stages of IC outlined by Neale (2016, 2021).

#### 2.5.1 Findings and Discussion

Two distinct themes were present in participant narratives explaining why DSA morbidity and mortality are so high in Middlesbrough: structural determinants and socio-cultural determinants; these themes can, in turn, be analysed through the theoretical lens of structural violence by identifying the specific policies driving them and their historical context. Structural determinants referred to factors related to social structures (e.g., economic and social policies, working conditions, and housing). Socio-cultural determinants referred to factors related to social norms and attitudes and individual behaviours (e.g., Gender roles and drug use behaviours). Participants did not provide explanations that identified exclusively structural or socio-behavioural determinants; rather, participant narratives contained elements of both themes to varying extents.

## 3 Structural determinants

Participants identified a number of structural determinants that they believed underpinned DSA morbidity and mortality in Middlesbrough. Chief among these factors were those that contributed to deprivation in the area, namely, deindustrialisation, austerity, and poor housing stock.

### 3.1 Deindustrialisation and austerity

Participants reported that prior to deindustrialisation Middlesbrough was, if not affluent, a place where working class people could make a decent living. It was believed by participants that the economic well-being of the town was tied to industry and the process of deindustrialisation ushered in a level of deprivation, caused primarily by a reduction in employment opportunities, low wages, and non-unionised employment, that had not been present in the community before. Participants believed that since the 1980s, work opportunities in Middlesbrough have been extremely limited and there was a severe level of poverty, with people struggling to meet their most basic needs like food, clothing, and heating their homes. Participants were correct in their belief that in recent years Middlesbrough has experienced above-average levels of unemployment. In 2022, the unemployment rate in Middlesbrough was 5.1%, higher than average for the North East (4.2%) and Britain as a whole (3.7%) (ONS, 2024a). Those who were in full-time employment in Middlesbrough in 2023 earned significantly less gross weekly pay (£554.7) than is average in Britain (£682.6) (ONS, 2024b). Middlesbrough was ranked as the most income-deprived local authority in England (out of 316) in 2019 (most recent data) (IOD2019, 2019). The below-average income and employment present in Middlesbrough are products of deindustrialisation. The loss of industrial jobs during the 1980s resulted in unemployment and low wages in affected areas like Middlesbrough which persists today, and new job growth has often been in low-wage sectors such as call centres and warehouses (Beatty and Fothergill, 2018). That the Government has consistently failed to alleviate the deprivation caused by deindustrialisation and has implemented policies that have exacerbated it, is an example of structural violence. Participants stressed the importance of the challenges presented by deprivation and felt that this was the most important factor driving DSA morbidity and mortality in their community; in this way, structural violence is at the root of DSA morbidity and mortality in Middlesbrough today.

“I can’t get far past deprivation to be honest with you. I think if you transplanted everyone in Middlesbrough down to some nice leafy part of Surrey and gave them the average income for people in that area, you’d see things change faster than you can imagine.”

The deprivation caused by deindustrialisation was believed to have set the stage for DSA morbidity and mortality in Middlesbrough. While deindustrialisation was not a uniquely British phenomenon and has been observed to have had a negative effect on communities around the world, government policy initiatives implemented by the Thatcher government in the 1980s amplified the already severe impacts of deindustrialisation. Government spending on welfare benefits was reduced, leaving recently unemployed industrial workers in Middlesbrough vulnerable to poverty. (Albertson and Stepney, 2020; Scott-Samuel et al., 2014). The loss of industrial jobs resulted in higher levels of unemployment and increased reliance on incapacity benefits, further straining public finances. The persistent economic inactivity and reliance on benefit in areas like Middlesbrough are direct consequences of the long-term effects of deindustrialisation which would be compounded by the austerity measures introduced in 2010 (Beatty and Fothergill, 2017). Austerity measures reduced local authority budgets by 30% between 2008 and 2015 and led to the closure of many public services (Bach, 2016). The worst-hit local authority areas – those mainly located in the North and including Middlesbrough - lost around four times as much, per adult of working age, as the authorities least affected by the cuts – found exclusively in the South and East of England (Beatty and Fothergill, 2018; Gray and Barford, 2018). This is an example of how place intersects with structural violence; while austerity reduced local authority budgets everywhere, the effects of this structural violence were greater in vulnerable communities like Middlesbrough.

Participants pointed to austerity as a specific turning point in the town’s history that had a significant negative effect on the service provision capacity of the town and the general quality of life for people living in the area. Many believed that since austerity, drug-related deaths had become more common. Participants attributed the increase in drug-related deaths post-austerity to the loss of specialist capacity for drug treatment.

“Focusing on drug services, we saw a dramatic increase in the number of deaths over that period of time. Rapid. So, austerity, or things linked to austerity, caused the drug-related death rates to rise and rise and rise and rise.”

Austerity was believed to have weakened the service provision landscape in the community and made it more difficult for stakeholders to help people vulnerable to DSA morbidity and mortality. Participants felt that services across sectors were overwhelmed and struggling to meet the level of demand. There was agreement amongst participants that the level of need in the community had increased, while the level of funding had reduced. Participants felt that the reduction in service capacity and increase in demand had led services to be driven by what they could afford to provide, rather than what their clients needed most. There are clear geographic patterns in austerity-related funding cuts, with northern local authorities losing more per working-age adult than their southern counterparts, which worsened existing regional health inequalities (Scott-Samuel et al., 2014). According to participants, many services had a waiting list, and the waiting list for mental health assessments on the NHS was far too long to be useful for people in acute need.

“When I was in Uni studying in social work the key message is “your patient, your service … they have to be your number one priority” but actually, the way the service is organized, and I don’t know what the answer is, actually we’re not needs-led, we’re resource-led.”

Austerity was also believed to have harmed the community as a whole, beyond its direct impact on drug-related deaths. Some of the harms of austerity were tangible, such as a loss of funding for youth provision and a reduction in funding for council services.

“[*Austerity*] had a massive impact on our communities. I can watch this unfold and watch what’s happening. There’s just nowhere for young people to go. We’ve got three youth clubs, in the whole of Middlesbrough. That’s not enough.”

Other impacts of austerity were less tangible and related to how stakeholders and the people they served viewed their community in relation to the central government. Participants explained that austerity showed that the central government (that being the various governments that implemented or maintained austerity since 2010) did not understand or care about the needs of Middlesbrough. Participants reported that the lack of interest in helping Middlesbrough fostered a sense of resentment and hopelessness within the town because people had seen that conditions were getting worse and believed that this would continue since the government was not interested in helping them.

“We live in a society where I don’t think they want to resolve [*issues in the community*] fully. I think the government that we have in power wants people that they deem as feckless in society. They don’t want people to be educated. They don’t want to be challenging the status quo. They don’t want to be challenging them and that disparity is getting greater and greater based on the last number of years of government. I don’t see it getting better anytime soon unless we have a change in government or a change in tracking in how we look at these socio-economic issues”.

Participants were acutely aware of the damaging effects that neoliberal economic policies of the 1980s and the 2010 austerity measures have had on Middlesbrough and believed the harm caused by these policies could not be ignored; this is an example of structural violence. The budget cuts and welfare reforms of the last 40 years in the UK have had an objectively harmful effect that has been empirically documented to have resulted in loss of life and increased suffering (Corris et al., 2020; Roscoe et al., 2021; Scott-Samuel et al., 2014). Given stakeholders’ awareness of the damaging effects that 40 years of policy decisions have had on Middlesbrough, it is perhaps unsurprising that they expressed resentment for the central government and the belief that the government does not care about the well-being of local communities. Resentment is a common response to deindustrialisation that has been observed in communities severely impacted by deindustrialisation from North America (Linkon, 2018), to Northern England (Webster, 2003), to Eastern Europe (Scheiring and King, 2023). Stakeholders reported that they, and residents of the area, have seen that conditions have deteriorated from the high experienced in the latter half of the 20th century, that the government has done little to slow the decline, and has, at times, made it worse; thus, they have no reason to believe that things will change going forward. The finding that deindustrialisation and austerity have given rise to a pessimistic worldview among stakeholders in Middlesbrough and the people they work with is consistent with research in deindustrialised communities around the world and highlights how structural violence affects the way that individuals view themselves in relation to their government. This finding also highlights that the effects of structural violence are specific to individual places. Austerity did not give rise to this pessimistic worldview everywhere; it did so in Middlesbrough because it occurred in the historical context of a deindustrialised community.

### 3.2 Poor housing stock

The housing stock in Middlesbrough was believed to be poor. Council estates have been privatised, a phenomenon that stems from the Housing Act 1980, also known as “right to buy”, and as a result most affordable accommodation comes from private landlords (Disney and Luo, 2017). Private accommodation in Middlesbrough was consistently described as low quality and dangerous. Participants reported that landlords would not house people with a wide range of criminal convictions that are common among people who have experienced homelessness or been evicted. Participants believed that housing providers would rather have empty housing units than house tenants perceived as difficult. Participants also stated that drug use and drug-related crime were common in private accommodation and that people living alone in shared accommodation were vulnerable to sexual abuse and exploitation and would be exposed to drug dealing and crime.

“The private rented sector especially but even some of the hostels and registered providers, some of the social landlords, I sort of, people who are placed in those accommodation options are surrounded by active drug use, drug dealing, bothering them, tempting them, manipulating them, crime, antisocial behaviour, all of these things … they’re dangerous places. Not the sort of place you’d want to live.”

According to participants, living in low-quality accommodation that was dangerous was distressing, so people sometimes turned to drugs or alcohol as a way of coping. Participants believed that living in low-quality accommodations also made it harder for people who were recovering from substance abuse issues to achieve and maintain sobriety because they were often surrounded by drug users and dealers. The effects of structural violence in Middlesbrough are also evident in the poor housing stock described by some participants. Right to Buy legislation facilitated the transfer of publicly-owned accommodation to private individuals in order to promote homeownership and the building of capital by the middle class (van Ham et al., 2013). Criticism of Right to Buy legislation is not new and the negative impacts are well documented (Beswick and Penny, 2018; Cooper et al., 2020); this study provides further evidence that this legislation continues to cause harm by exacerbating DSA morbidity and mortality in vulnerable communities today.

### 3.3 Responses to deprivation - distress and hopelessness

Participants believed that deprivation caused two emotional states, distress, and hopelessness, that contribute to DSA morbidity and mortality. Although similar, distress and hopelessness were distinct, and participants believed that they were not the same as having a diagnosable mental illness. Distress, as participants used the term, referred to an acute state that occurred when a person became overwhelmed by their social circumstances (e.g. the loss of a job, inability to pay the bills). Participants saw distress as more closely linked to suicide than to drug and alcohol-related deaths. Hopelessness, as participants used the term, referred to a worldview that developed because of long-term exposure to deprivation; this worldview was characterised by the belief that one would always live in deprivation and that they could not expect their situation to ever improve.

“These are probably not people who are acutely, what we would call acutely mentally ill. They’re not suffering from like an acute psychotic episode, they’re not deeply depressed, or those kinds of things. They’re just people who are extremely distressed … often we’re seeing people and they’re not mentally ill, their lives are just shit.”

Given the above-average prevalence of deprivation and the high unemployment rate in the area (IOD2019, 2019) long-term exposure to deprivation is particularly common in Middlesbrough; this is a reflection of a broader North-South divide in England. The North-South divide refers to the longstanding geographic health and wealth inequalities between the North and South of England, the root causes of which are generally acknowledged to be political in nature (Bambra et al., 2014; Bambra, 2016) and is itself a manifestation of structural violence. The North, historically reliant on industrial work, faced severe economic decline with the shift to a post-industrial economy. In contrast, the Southeast, particularly London, benefited from the growth of well-paid jobs in finance and other sectors, leading to significant regional disparities (Hudson, 2005; Martin and Sunley, 2023). Specific policies, such as those of the Thatcher administration in the 1980s and the concentration of investment in London and the Southeast, exacerbated these inequalities (Hudson, 2005; Martin and Sunley, 2023). These disparities are a manifestation of structural violence and are emblematic of how the effects of structural violence are not applied evenly; they affect some places, like Middlesbrough, more than others. Despite efforts by the central government between 2000 and 2010 to reduce regional social and health inequalities (Whitehead, 2007), and the Levelling Up agenda, the latest policy initiative that sought to address geographical inequalities, (Fransham et al., 2023), the economic and health gaps between the North and South have grown in England since 2010 (Taylor-Robinson et al., 2019) and were further exacerbated by the COVID-19 pandemic (Bambra et al., 2020; Robinson et al., 2020). Participants believed that since people were resigned to living in deprivation, which itself was a product of the structural violence of regional wealth inequalities, they had become disempowered and had no aspirations for the future. Additionally, Middlesbrough is geographically isolated, lacking convenient access to regional economic hubs such as the cities of Newcastle Upon Tyne (32 miles to the north) and York (43 miles to the south). Participants believed that the town’s geographic isolation exacerbated deprivation in their areas since people lacked access to work and educational opportunities available in larger cities. These findings demonstrate how the structural violence of regional economic inequalities and the place specific factors combine to create DSA morbidity and mortality in deprived areas like Middlesbrough.

“People just accept that its fait accompli that that is what’s going to happen, and they have no realistic expectation that it can ever be any different, that they can ever change. That is really sort of a tough place to be starting when we’re trying to engage and promote positive behaviour change.”

When people were distressed by acute social circumstances or feel hopeless because of long-term exposure to deprivation, participants explained they would often turn to drugs, alcohol, or self-harm as a coping mechanism. Drugs and alcohol were seen as similar coping mechanisms that allowed one to “numb out” the distress and hopelessness for a time.

“I think [*drugs are*] a means of escape and numbing that reality of what their life looks like or the pain that they’re caught up, whatever it might be, but yeah. I guess that’s probably the best word I can use to describe it, it’s the numbing of the here and now.”

While drugs and alcohol were seen as a way of numbing pain or self-medicating, suicide and self-harm were viewed as a form of escapism or a cry for help. Participants felt that many people who attempted suicide did not want to die necessarily, but they desperately wanted to change their situation and did not know how to ask for help.

“Most people actually don’t want to die. They just want, the people who tell us they want to die, they’re going to kill themselves [*sic*], they want the, they want help and comfort from us, rather than to die. The people that die, they don’t tell us. They just go ahead and do it.”

There is notable similarity between the phenomena of distress and hopelessness as identified by participants in this study and Case and Deaton’s concept of despair. While Case and Deaton’s (2020) definition of despair captures, in part, the phenomenon identified by stakeholders in this study, the findings of this study indicate that the leading role that despair is given in the term ‘deaths of despair’, and its classification as a cause of these deaths, is misplaced. The hopelessness is arising due to structural level factors, which are themselves a product of structural violence. While Case and Deaton (2020) acknowledge that social forces, including economic instability, are responsible for increasing rates of despair among middle age non-Hispanic whites in the US, their decision to place despair in their label for DSA mortality and their reference to it as a cause of these deaths, is indicative of an overemphasis of the role that despair plays in leading to deaths by these causes. Distress and hopelessness, or despair, are not ‘causing’ DSA morbidity and mortality in Middlesbrough, rather; they are the results of deprivation, which is itself simply one step on the pathway from structural violence to DSA morbidity and mortality.

## 4 Socio-cultural determinants

Some participants identified a number of socio-cultural determinants of DSA morbidity and mortality that affect Middlesbrough and the UK as a whole. These socio-cultural determinants included social attitudes and behaviours surrounding drug use, parenting standards, and changes to Middlesbrough’s communal identity.

### 4.1 Parenting standards and the normalisation of drug use

Some participants felt that parents have become too ‘soft’ on their children. These participants explained that parents in Middlesbrough no longer disciplined their children, and they prioritised doing what was easy over doing what was right for their child’s well-being. According to participants, a lack of discipline allowed children to do whatever they wanted, which lead to children misbehaving and using drugs. When children were caught using drugs or committing crimes, parents did not discipline them because they have become too soft. Participants felt that because parenting standards had slipped, the current generation of young people had low moral standards and poor self-regulation. Younger generations were seen to routinely engage in criminal behaviours, such as drug use and underage use of vaping products because their parents allowed it. Young people’s ‘out of control’ behaviour was believed to continue into adulthood and leave them without a clearly defined moral compass. Some participants believed that there is a cohort of young people, particularly men in Middlesbrough who have been allowed to grow up uneducated, undisciplined, and were now incapable of being functioning members of society.

“We have a cohort at the minute that seems to be predominantly young men. They have absolutely no skills at all. Absolutely no skills. They can’t keep a phone. They don’t know how to do a gas bill. They shit in wardrobes. They don’t know how to feed themselves. They have absolutely no liveable skills.”

Participants believed that social attitudes surrounding drug use had changed and that as a result more people were choosing to use drugs and developing addiction. These participants reported that drug use used to be stigmatized, but it was increasingly acceptable in Middlesbrough. Young people chose to use drugs over alcohol and the use of marijuana and/or cocaine was now considered normal, and their parents would not stop them. Participants believed that cocaine and marijuana use were harmful to one’s mental health and that the increasing use of these drugs was responsible for a decline in mental health and a corresponding rise in suicides. Cocaine was believed to be linked to suicide in young men; participants explained that young men used cocaine to experience a high and then attempted suicide in the ‘low’ that ensues after a cocaine binge.

“No, suicides are not on the up because we live in deprived areas. Suicides are on the up because people are doing drugs and sniffing cocaine.”

Participants believed that as drug use had become normalised, parents increasingly used drugs, particularly marijuana and cocaine, in front of their children. Participants believed that when parents used drugs in front of their children it taught them that it is okay to use drugs; this in turn encouraged young people to start using drugs and furthered the normalisation of drug use.

“There are a lot of children smoking cannabis in this town. It’s probably because it’s accepted at home. Their parents are smoking at home, and they give it to their kids. It’s so widely accepted that people have almost forgotten it’s a controlled drug, and rightly so as well.”

Participants’ accounts that drug use had become increasingly common in their community as a result of changes in parenting standards are not consistent with empirical evidence surrounding the prevalence of drug use. While rates of drug-related deaths in England have increased in recent years, there is little evidence that people are using drugs at higher rates than in previous decades (Augarde et al., 2022). While marijuana remains the most popular illicit drug used in England, rates of marijuana use among young people have steadily decreased over the last 20 years (NHS Digital, 2022). While there has been a slight increase in the rate at which people use drugs in England since 2012, the rate is still below the historical average since data collection began in 1995 and is lower than it was in any year before 2012 (NHS Digital, 2022). Possible explanations for the increasing rates of drug-related deaths nationally include that there is an ageing cohort of drug users for whom drug use is becoming more dangerous, an increase in the rate of polysubstance use (the use of multiple drugs at one time, which creates the risk of dangerous drug interactions), and suboptimal dose and duration of opioid agonist treatments, not by an increase in the number of people who use drugs (Kimber et al., 2019; van Amsterdam et al., 2021).

Given the lack of empirical evidence for an increase in the rate at which people use drugs in England, it is difficult to say that changing parenting standards are causing more drug use. Concerns about a perceived decline in the morals and values that parents instil in their children have an exceptionally long history in British society. In the 18th century, William Hogarth, an artist and social critic, depicted the moral decay he perceived in urban society through his works *Gin Lane* and *Beer Street* (Nicholls, 2003). In the 19th century, William Booth founded the Salvation Army to promote Christian morality and social reform to address what he saw as the erosion of Christian values amongst the poor (Hattersley, 2017). In the 20th century, Margaret Thatcher spoke of the need for a moral rejuvenation of Britain to address what she described as fecklessness and immoral behaviour amongst the working class (Tomlinson, 2021). Participants’ concern that changes in parenting standards have degraded the morals and values of British society follow a well-established precedent of laying the blame for this perceived change at the feet of impoverished people living in deprived urban conditions without the support of empirical evidence. Concerns about degrading morality and changing parenting standards absolve policymakers of responsibility for the harms attributable to decades of neoliberal economic policies that have had a measurably harmful effect on health and wellbeing in places like Middlesbrough and allow the continued implementation of such policies (Garthwaite, 2011; Schrecker and Bambra, 2015); in this way, these beliefs are themselves part of the cycle of structural violence.

It is similarly unclear that the normalisation of drug use has a significant impact on deaths of despair. Normalisation theory, advanced by Measham et al. (1994) explained how drugs become normalised within sub-cultures and broader society (Pennay and Measham, 2016). Measham et al. cited increasing accessibility of drugs, increasing rates of drug experimentation during youth, high levels of drug knowledge, and the cultural accommodation of certain drugs by non-drug users (e.g. in popular culture) as evidence that drug use had become socially acceptable and culturally embedded in society (Parker et al., 1995; Parker, 1998; Measham et al., 1994). Since the normalisation theory was advanced, researchers have further explored the process of drug normalisation; some of this research supports normalisation theory (Pearson, 2001, Simpson et al., 2007), while some has found that other theoretical approaches better explain increasing rates of drug use (Gourley, 2004; Shildrick, 2002).

Participants’ belief that many more people are using drugs in Middlesbrough than in previous decades may be a product of increased visibility of drug users. The use of crack cocaine, ketamine, and new psychoactive substances (such as synthetic cannabis, street name “spice”) increased between 2015 and 2020 (ONS, 2022). Use of these substances, particularly synthetic cannabis, is associated with psychiatric symptoms such as agitation, paranoia, and psychosis (Tait et al., 2016). It is therefore possible that increasing rates of use of these drugs have led to an increase in highly visible antisocial behaviour from people who use these drugs, creating the impression that more people are using drugs than was previously the case. It is also possible that while rates of drug use are still below historical averages, the visibility and disruptive nature of drug use has increased due to the influence of structural forces. As a result of austerity, police budgets have been cut (NAO, 2018), youth services have been closed (Davies, 2019), drug treatment services have faced budget reductions and closures (Roscoe et al., 2021), and access to mental health services has declined (Cummins, 2018). While rates of drug use have not increased, a combination of these structural forces may be forcing drug use into the public eye. If this is the case, participants have correctly identified a problem (that drug use is an increasingly visible and disturbing problem in their community) but misidentified the cause. This finding suggests that the effects of structural violence in the form of austerity can be misattributed to cultural forces.

### 4.2 Communal identity

In addition to the post-2008 austerity measures, deindustrialisation and the economic policies of the 1980s caused a loss of identity from which the community has still not recovered. Participants reported that people in the community still viewed Middlesbrough as an industrial town because nothing had replaced industry as the driving economic force. The centrality of industry to Middlesbrough’s identity was evident when participants were asked to describe the area; stakeholders frequently described Middlesbrough with words such as “industrial” and “traditional”. Even the colloquial name for people from Middlesbrough and the surrounding communities of Teesside “Smoggies”, a reference to the smog produced by heavy industry, harkens back to the town’s industrial legacy. While participants believed that industry was still central to Middlesbrough’s identity, they acknowledged that work in the industrial sector had largely left the area. Participants described the disconnect between Middlesbrough’s communal identity and the economic reality in stark terms. Middlesbrough was described as “a shell of a place” and “the land of the living dead”. Some participants felt that establishing a new source of communal identity for the town was an important step in establishing a prosperous future.

“There needs to be progress made on “what does Middlesbrough become?” Otherwise, we’ll always be stuck on “what was Middlesbrough?”

Trauma is often conceived as something that happens to individuals after experiencing a distressing event; however, participants’ accounts provide evidence that another form of trauma may be present in Midlesbrough. Collective trauma refers to the psychological distress that a group, such as a community or culture, experiences in response to a shared trauma (Hirschberger, 2018). While collective trauma is often observed in communities that have experienced violent conflict or been victimised by racial or ethnic violence (Li et al., 2022), deindustrialisation has been conceived of as a form of structural violence that results in collective trauma (Lawson, 2020; Clark, 2023). Unlike individual memories of traumatic experiences, collective trauma is remembered by the community as a whole and may be remembered by individuals who did not directly experience the traumatic event (Hirschberger, 2018); this process is known as transgenerational transmission of trauma (Volkan, 2001).

The concepts of transgenerational and collective trauma can be helpful in understanding how the structural violence of deindustrialisation, a process which peaked in Middlesbrough in the 1980s, continues to have a profound impact on the identity of the town today. The identity of a community is socially constructed and transferred across generations (Emery, 2019). The collective trauma of deindustrialisation disrupts the intergenerational transfer of identity as older generations struggle to articulate communal identity amid changing social and economic circumstances (Bright, 2012). Younger generations in deindustrialised areas, like Middlesbrough, are left with identities and social expectations (ie. that an adult man without further education will be able to secure full-time, long-term employment and provide for a family) constructed around historical industries that are unable to be achieved in current post-industrial labour market conditions. The division between what is expected of people as members of a community and what is realistically attainable has been identified by sociologists as a source of shame and a sense of collective loss (Walkerdine, 2010; Walkerdine and Jimenez, 2012). In Middlesbrough, this fractured sense of identity helps to explain why there is a connection between the structural violence of deindustrialisation and the feeling that participants described as hopelessness. Older generations in Middlesbrough experienced deindustrialisation and the entailing losses first-hand, while younger generations have been raised with a set of cultural expectations that they cannot meet.

“My partner was saying, he explained it like the men would go working down the pit. They’d come back, go to the pub, slap their wife about a bit, and get up and go to work. I think we still have that kind of attitude in a lot of men. I think it’s been passed down through the generations, that expectation. The culture is very much still there. So, if you think some of these men, they don’t have jobs, they can’t get a job. They’re not able to, not fulfilling their purpose to provide. It’s a recipe for suicide. They’re lacking that purpose.”

The gap between what is culturally expected of people in Middlesbrough and what is realistically achievable may partially explain why Middlesbrough has worse DSA-related outcomes than other places in the North East. Given that one of Middlesbrough’s chief industries was steel production and that former steel-producing areas have been observed to have a stronger connection between their industry and their collective identity than other industrial regions (Rhodes, 2013; Zukin, 1993), it is possible that the failure to achieve cultural expectations is felt more viscerally by people in Middlesbrough than in other deindustrialised places.

## 5 Conclusion

**Fig. 2:**
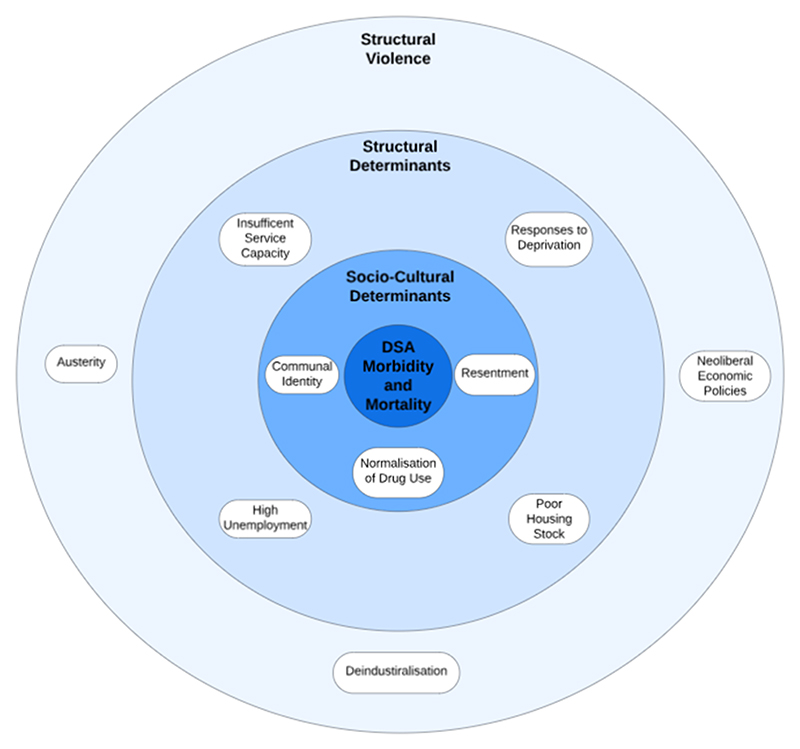
Narrative map of themes driving DSA morbidity and mortality in Middlesbrough.

Structural violence explains how political and economic systems and policies perpetuate violence and cause significant harm to individuals or groups of vulnerable people within a society (Galtung, 1969). Participants’ narratives outline how structural violence in the form of austerity and the neoliberal economic policies of the 1980s has created determinants at the structural and socio-cultural levels that ultimately drive DSA morbidity and mortality in Middlesbrough today. Fig. 2 presents a concept map of themes present in participants’ narratives.

It is clear that the factors driving DSA morbidity and mortality are more complex than may initially be assumed. Neither deindustrialisation, the neoliberal economic policies of the Thatcher administration, nor austerity is solely to blame for the above-average prevalence of DSA morbidity and mortality in Middlesbrough; rather, all of these factors had a compounding harmful effect that have created the environment in which deaths from these causes arise. A theoretical framing of the determinants of DSA morbidity and mortality as a product of structural violence provides a clearer understanding of how socio-cultural, and structural determinants constitute a complex web of influences and underscores the need for approaching DSA morbidity and mortality as issues rooted in social injustice. DSA Morbidity and mortality are a manifestation of broader social inequality, and interventions seeking to prevent deaths from these causes must address the upstream forces, such as income deprivation, housing, and austerity, that have given rise to the conditions in which people consider initiating DSA behaviours.

Our findings suggest that stakeholders in Middlesbrough viewed deaths of despair somewhat differently than they have been traditionally conceived in other contexts. In the US, Case and Deaton (2017, 2020) framed deaths of despair as those that predominantly impact people of middle age whereas stakeholders in our study spoke most often about young adults and adolescents. Case and Deaton proposed that changes in social norms and the US labour market caused cumulative disadvantage from one birth cohort to the next had given rise to a sense of despair and ultimately increased the likelihood of DSA mortality (Case and Deaton, 2017). In this study, we have provided evidence that in Middlesbrough DSA morbidity and mortality are themselves a manifestation of existing regional health and wealth inequalities. There is some evidence to support this view, as recent research has described how deaths of despair have manifested differently in populations other than middle-aged non-Hispanic Whites in the (Aspholm, 2022; DeVerteuil, 2022). The differences between our findings and the established deaths of despair literature in the US supports the view that deaths from these causes look differently in different contexts and populations. Researchers examining deaths of despair outside of the US and outside of the traditionally affected demographic groups should be mindful that populations other than those identified by Case and Deaton may experience a similar phenomenon, although their trajectories may vary.

Existing research on the determinants of deaths of despair has largely used quantitative methods to investigate population-level factors associated with increased mortality risk from these causes (Beseran et al., 2022). The narrative map in Fig. 2 offers a novel approach to understanding how structural violence has created the context in which structural and socio-cultural determinants intersect to produce DSA morbidity and mortality in Middlesbrough. This narrative map was formulated inductively and is grounded in participants’ real-world experiences working with and on behalf of people in Middlesbrough. It is also consistent with existing empirical literature discussed above that has linked factors such as the collective trauma of deindustrialisation, resentment towards government, and deprivation to these outcomes. The consistency of findings with empirical literature indicates that this narrative map is a valid way of conceptualising DSA morbidity and mortality in deindustrialised communities within the UK. Future qualitative research should investigate how the structure of the narrative changes in different contexts with different populations. In interpreting the findings presented in this paper, it is important to consider the sample from which they are derived. The sample included stakeholders from a broad range of professional sectors, which ensured the findings represented a diverse set of professional experiences and viewpoints. Despite this, there was little variance in participants’ opinions on the determinants of DSA morbidity and mortality (i.e. most participant narratives contained elements of both socio-cultural and structural determinants to varying degrees), suggesting that participants’ perspectives were not heavily influenced by their professional background. Most of the participants in this study had some degree of advanced education, with several having studied at the postgraduate level. All, by nature of their professional roles, had experience interfacing with government policy and had first-hand knowledge of how policy decisions and government directives influence service delivery. Further qualitative work with community members in Middlesbrough will reveal whether, and to what extent, people without such experience understand the problems of DSA morbidity and mortality in their community in relation to government policy. Additionally, the recruitment methods used in this study relied on snowball sampling and professional networks in Middlesbrough, which may have inadvertently restricted participation to a group of the most networked individuals; there may be groups outside of this structure which have unique insight who were not reached by recruitment efforts.

Stakeholders participating in this study identified structural and socio-cultural determinants that they believe drive DSA morbidity and mortality in their community. In this paper, we have made the case that the determinants identified by stakeholders are themselves a product of structural violence perpetuated by a series of neoliberal economic policies spanning the last 40 years. On a more practical level, our findings suggest that attempts to interrupt the pathways leading to DSA morbidity and mortality in deindustrialised communities must be multifaceted and seek to address the broader social determinants of health that underly drug, suicide, and alcohol-specific mortality.

## Supplementary Material

**Appendix A.Supplementary data**

Supplementary data to this article can be found online at https://doi.org/10.1016/j.healthplace.2024.103346.

## Figures and Tables

**Table 1:** Participant demographic information.

Category	Number of Participants
**Gender**	
Male	6
Female	7
**Age**	
25−34	1
35−44	1
45−54	7
55−64	4
**Highest Level of Education**	
Higher or secondary or further education (A-levels, BTEC, etc.)	4
University	6
Postgraduate degree	2
Prefer not to say	1
**Years in Current Professional Role**	
Less than 1 Year	4
1−2 Years	3
2−5 Years	0
5−10 years	1
More than 10 Years	4
Prefer not to say	1

## Data Availability

The data that has been used is confidential.
